# Effect of sunlight exposure on cognitive function among depressed and non-depressed participants: a REGARDS cross-sectional study

**DOI:** 10.1186/1476-069X-8-34

**Published:** 2009-07-28

**Authors:** Shia T Kent, Leslie A McClure, William L Crosson, Donna K Arnett, Virginia G Wadley, Nalini Sathiakumar

**Affiliations:** 1 Department of Epidemiology, School of Public Health, University of Alabama at Birmingham, 1665 University Blvd, Birmingham, Alabama, USA; 2 Department of Biostatistics, School of Public Health, University of Alabama at Birmingham, 1665 University Blvd, Birmingham, Alabama, USA; 3 National Space Science and Technology Center, NASA Marshall Space Flight Center, 320 Sparkman Drive, Huntsville, Alabama, USA; 4 Department of Medicine, University of Alabama at Birmingham, 1808 7th Avenue South Birmingham, Alabama, USA

## Abstract

**Background:**

Possible physiological causes for the effect of sunlight on mood are through the suprachiasmatic nuclei and evidenced by serotonin and melatonin regulation and its associations with depression. Cognitive function involved in these same pathways may potentially be affected by sunlight exposure. We evaluated whether the amount of sunlight exposure (i.e. insolation) affects cognitive function and examined the effect of season on this relationship.

**Methods:**

We obtained insolation data for residential regions of 16,800 participants from a national cohort study of blacks and whites, aged 45+. Cognitive impairment was assessed using a validated six-item screener questionnaire and depression status was assessed using the Center for Epidemiologic Studies Depression Scale. Logistic regression was used to find whether same-day or two-week average sunlight exposure was related to cognitive function and whether this relationship differed by depression status.

**Results:**

Among depressed participants, a dose-response relationship was found between sunlight exposure and cognitive function, with lower levels of sunlight associated with impaired cognitive status (odds ratio = 2.58; 95% CI 1.43–6.69). While both season and sunlight were correlated with cognitive function, a significant relation remained between each of them and cognitive impairment after controlling for their joint effects.

**Conclusion:**

The study found an association between decreased exposure to sunlight and increased probability of cognitive impairment using a novel data source. We are the first to examine the effects of two-week exposure to sunlight on cognition, as well as the first to look at sunlight's effects on cognition in a large cohort study.

## Introduction

It is widely accepted that climate and season affect psychological characteristics [1,2]. Recent research has shown that serotonin and melatonin regulation, mechanisms that are involved in the relationship between sunlight and light therapy on mood, are also involved in cognition, which suggests that cognitive function may also be influenced by light [3-5]. Melatonin, serotonin and other mechanisms involved in circadian rhythms are associated with cognitive functioning, and are regulated by the suprachiasmatic nuclei (SCN), which are susceptible to the effects of differing intensities and patterns of environmental illumination [6]. However, the effect of sunlight and light therapy on cognitive function has not been adequately studied. This study aimed to explore if sunlight exposure, measured by insolation (the rate of solar radiation received in an area), is associated with cognitive impairment. In addition, examined the role of season in this relationship. This study was conducted using baseline data from a large prospective study, the REasons for Geographic And Racial Differences in Stroke (REGARDS) Study, and National Aeronautics and Space Administration (NASA) satellite and ground data. We hypothesized that lower levels of sunlight exposure at participants' residences would be associated with increased rates of cognitive impairment. This study was the first to examine the effects of two-week exposure to natural sunlight on cognition, as well as the first to look at solar effects on cognition in a large cohort study.

## Methods

### Participants

The present study consisted of participants from the REGARDS study, which has been described in detail elsewhere [7]. In brief, REGARDS is a longitudinal study being conducted to determine relationships between various risk factors and the incidence of stroke. The participants are aged 45 and older and sampled from the 48 conterminous United States. Study participants were oversampled from the "Stroke Belt", a high stroke mortality region consisting of the 8 southeastern states of Arkansas, Louisiana, Tennessee, Mississippi, Alabama, Georgia, North Carolina, and South Carolina. The sample population was particularly oversampled from the "Stroke Buckle", a region with even higher stroke mortality along the coastal plains of Georgia, North Carolina, and South Carolina. Within each region the planned recruitment included half white and half African-Americans (actual: 41% African-American, 59% white). Planned recruitment within each race-region strata was half male and half female (actual total recruitment: 45% male, 55% female). At baseline, a telephone interview was conducted that recorded the patient's medical history, demographic data, socioeconomic status, stroke-free status, depression, and cognitive screening. An in-home exam was conducted recording height, weight, and blood pressure. All participants provided written informed consent, and the study was approved by the Institutional Review Board for Human Subjects at the University of Alabama at Birmingham, as well as all other participating institutions.

### Sunlight Exposure (Insolation) Assessment

Sunlight exposure was measured using data values prepared and provided by NASA's Marshall Space Flight Center. Solar radiation values were obtained for 2003 to 2006 from the North American Regional Reanalysis (NARR), an assimilated data product produced by the National Center for Environmental Prediction (NCEP), a division of the U.S. National Weather Service. The product, including information from satellites and ground observations, was compiled on a grid with a 32 km resolution over North America and matched to each participant by the geocoded residence obtained from the REGARDS database. Solar radiation in Watts/meters^2 ^(W/m^2^), a measure of the instantaneous solar energy reaching the Earth's surface, was assessed 8 times a day at 3-hour intervals for each residence starting at 1:00 AM Pacific Standard Time (PST). A daily integral of solar radiation was calculated; this is referred to as insolation and has units of kilojoules per meters^2 ^per day (KJ/m^2^/day). As a point of reference, under clear skies in late spring or early summer, a typical daily insolation value in the central U.S. is approximately 25,000–30,000 KJ/m^2^. In late fall or early winter, a typical daily value is approximately 8,000–10,000 KJ/m^2^.

The current residence from the original recruitment file plus updated information from participant at time of scheduling in-home exam was used to geocode each participant. Geocoding of the participants was performed using SAS/GIS batch geocoding. Information obtained from SAS/GIS with 80% accuracy or greater was utilized. Using a subset of the data, we validated the results from the SAS/GIS procedure against a commercially available program http://www.geocode.com using the Haversine formula, and found there to be high agreement between the two algorithms [8]. For those with a SAS/GIS accuracy of 80% or greater, the difference between the latitudes given between the two programs had a mean of 0.23 kilometers and a maximum of 0.95 kilometers.

### Cognitive Assessment

A six-item screener questionnaire was used to evaluate global cognitive status by assessing short-term recall and temporal orientation [9-11]. As REGARDS has done in other studies, the score of this screener was dichotomized into an outcome of cognitively impaired or intact. A score of four or fewer correct responses out of the six questions indicated cognitive impairment. Callahan et al. 2002 validated the screener in both a community-based population of 344 black adults aged 65 or older and a population of 651 subjects who were referred to the Alzheimer's disease Center (16% black). Results from the community-based sample found that for a six-item screener score of 4 or fewer, using clinically confirmed cognitive impairment as the gold standard, the sensitivity was 74% and specificity was 80% [10]. The instrument was based on and validated against the Mini-mental State examination [11]. It was also validated against other cognitive measures and diagnoses of both dementia and non-dementia cognitive impairment [10].

### Participant Selection

A total of 19,853 participants without previous stroke were enrolled in the study at the time of this analysis (December 1, 2006). Of these, 3,020 patients with poor geocoding (less than 80% accuracy) and a further 33 patients who were missing cognitive scores were excluded, leaving 16,800 participants. Due to data missing in any of the potential confounders, 3,253 of the 16,800 participants were excluded during model selection. Once the model selection was completed, only 2,326 of the 16,800 participants were excluded due to missing covariates selected for the final multivariable model. Chi-squared and t-tests were used to measure differences of the 5,379 excluded and 14,474 included subjects in the final model.

### Statistical Analyses

We analyzed insolation from the day the six-item screener was administered and the preceding two weeks. Insolation measurements were analyzed as continuous variables and categorical variables (by 5,000 KJ/m^2^/day increments).

Due to prior evidence regarding relationships with cognitive function, we considered the following as potential confounders: sex, geographic region (stroke belt, stroke buckle, or non-stroke belt), population density (urban, suburban, and rural), income (less than $20,000, $20,000 to $34,999, $35,000 to $74,900, or $75,000 and more), education (less than high school, high school diploma, some college, or college diploma), race (black or white), smoking (current, past, or never), alcohol use (never used or ever used), Body Mass Index (BMI) (underweight, normal, overweight, or obese), hypertension status (systolic blood pressure ≥ 140, diastolic blood pressure ≥ 90 or self-reported use of hypertension medications), high cholesterol (cholesterol >240), diabetes status (fasting glucose ≥ 126, non-fasting glucose ≥ 200, or self-reported diabetes medications), exercise (weekly or less than weekly), depression status based on the Center for Epidemiologic Studies Depression Scale (CES-D) scale, physical function as measured by the 100 point scale Physical Components Summary (PCS) in the 12-item Short Form (SF-12), season of phone interview (spring, summer, fall, or winter), and age in years (45–54, 55–59, 60–64, 65–69, 70–74, 75–79, or 80 or more) [12,13].

T-tests, chi-squared tests, and correlation tests were used to determine preliminary relationships between insolation, cognition, and the covariates. Cochran-Mantel-Haenzel (CMH) chi-squared tests were used to determine if ordinally categorized insolation had relationships with categorical predictors. Logistic regressions were used to model the association between insolation and cognition. Backwards elimination was used to build the final multivariable model. Covariates whose relationships with cognitive function carried p-values over 0.10 were not considered for inclusions in the multivariable model. Due to the REGARDS sampling methods, the variables race, region, and sex were included in the multivariable model regardless of statistical significance. All interactions of the remaining covariates with insolation in the final models were considered. For any significant interactions the predicted probabilities and odds ratios (ORs) of cognitive impairment with their 95% confidence intervals were calculated. Since other factors related to seasonality besides insolation may be related to cognitive function, such as temperature, activity level, allergies, and stress, the final model was run both with and without season as a covariate [1,14-17].

Finally, the cognitive screener was divided into two components, the three points that measure short-term recall and the three points that measure temporal orientation. Each of these components was used to explore individual relationships with insolation. Univariate relationships were analyzed using chi-square tests and multivariable relationships were evaluated by taking the final logistic regression model obtained above and replacing the summary measure of cognitive impairment with each of the individual components of the six-item screener. For these analyses, the three point component measures were dichotomized, with one missing point indicating a deficit in either orientation or recall. The measures were also analyzed continuously, so that each component would be equal to the number of points obtained (0, 1, 2, or 3).

## Results

Continuous two-week insolation (p = 0.005) but not same-day insolation (p = 0.332) differed significantly by cognitive status (data not shown). Table 1 presents the baseline characteristics overall and by cognitive status. Sex, education, age, income, population density, season, diabetes status, hypertension status, depression status, PCS, alcohol usage, and weekly exercise all differed significantly by cognitive status (all p-values < 0.05; Table 1). CMH chi-squared tests indicated that gender, age, region, population density, season, BMI, PCS-12, and weekly exercise had dose-response relationships with ordinally categorized insolation, but education, diabetes, hypertension, high cholesterol, smoking (data not shown), and depression (Table 2) did not. Dose-response relationships of income (p = 0.0956) and alcohol use (p = 0.0650) with insolation were marginal (data not shown).

**Table 1:** Demographic, medical, and lifestyle characteristics by cognitive status

Characteristics	All Subjects (N = 16800)	Missing	Intact Cognitive Status (N = 15421; 92%)	Impaired Cognitive Status (N = 1379; 8%)	p-value
	**N (%)**	**N**	**N (%)**	**N (%)**	

**Demographics**					

Male	6657 (40)	0	6033 (39)	624 (45)	**<0.0001**

					

Education		19			

Less than High School	2081 (12)		1719 (11)	362 (26)	

High School	4404 (26)		3978 (26)	426 (31)	**<0.0001**

Some College	4525 (27)		4230 (27)	305 (22)	

College Diploma	5761 (34)		5480 (36)	281 (20)	

					

Age		5			

Less than 55 years	2077 (12)		1982 (13)	95 (7)	

55 to 59 years	2908 (17)		2756 (18)	152 (11)	

60 to 64 years	3197 (19)		3009 (20)	188 (14)	**<0.0001**

65 to 69 years	3117 (19)		2870 (19)	247 (18)	

70 to 74 years	2418 (14)		2172 (14)	246 (18)	

75 to 79 years	1765 (11)		1536 (10)	229 (17)	

80 or more years	1313 (8)		1091 (7)	222 (16)	

					

Income		2171			

Less than $20,000/year	3097 (21)		2698 (20)	399 (35)	

$20,000–35,000/year	4017 (27)		3633 (27)	384 (33)	**<0.0001**

$35,000-$75,000/year	4968 (34)		4678 (35)	290 (25)	

Over $75,000/year	2547 (17)		2467 (18)	80 (7)	

					

Region		0			

Non Belt/Buckle	7850 (47)		7211 (47)	639 (46)	0.13

Stroke Belt	6095 (36)		5567 (36)	528 (38)	

Stoke Buckle	2855 (17)		2643 (17)	212 (15)	

					

Population Density		0			

Urban	13532 (81)		12365 (80)	1167 (85)	

Mixed	1678 (10)		1571 (10)	107 (8)	**0.0003**

Rural	1590 (9)		1485 (10)	105 (8)	

					

Season		0			

Spring	3610 (22)		3260 (21)	350 (25)	**<0.0001**

Summer	6439 (38)		5998 (39)	441 (32)	

Fall	3410 (20)		3181 (21)	229 (17)	

Winter	3341 (20)		2982 (19)	359 (26)	

					

**Medical Factors**					

BMI		238			

Underweight	212 (1)		189 (1)	23 (2)	

Normal	3973 (24)		3651 (24)	322 (24)	0.26

Overweight	5970 (36)		5462 (36)	508 (38)	

Obese	6407 (39)		5906 (39)	501 (37)	

					

Diabetic	3527 (22)	623	3150 (21)	377 (29)	**<0.0001**

					

Hypertensive	9854 (59)	73	8943 (58)	911 (67)	**<0.0001**

					

High Cholesterol	1740 (10)	80	1595 (10)	145 (11)	0.85

					

Depressed	1877 (11)	155	1624 (11)	253 (18)	**<0.0001**

					

PCS-12 (mean, stddev)	46.1 (10.6)	0	46.3 (10.5)	44.0 (11.0)	**<.0001**

					

**Lifestyle Factors**					

Never Used Alcohol	5129 (31)	0	4606 (30)	523 (40)	**<0.0001**

					

No weekly exercise	5948 (36)	234	5359 (35)	589 (44)	**<0.0001**

					

Smoking		67			

Current	2445 (15)		2227 (15)	218 (16)	0.18

Past	6609 (40)		6055 (39)	554 (40)	

Never	7679 (46)		7078 (46)	601 (44)	

stddev = standard deviation; BMI = Body Mass Index; PCS = Physical Components Summary

P-values for categorical variables provided from a chi squared test statistic and for continuous variables provided from a scatterthwaite t-test statistic calculated for each variable by cognitive status.

P-values in **bold **indicate values that are significant at α = 0.05.

**Table 2:** Crude logistic univariate relationships of depression with two-week insolation

Characteristics	OR (95% CI)
**Primary Variable of Interest**	

2 week solar radiation (by 5,000 KJ/m^2^/day)	

<10,000 J/m^2^	1.14 (0.91–1.42)

10,000–15,000 J/m^2^	0.94 (0.81–1.09)

15,000–20,000 J/m^2^	0.87 (0.75–1.01)

20,000–25,000 J/m^2^	0.87 (0.76–1.00)

>25,000 J/m^2^	1.00 (Referent)

	CMH chisq p = 0.6054

OR = odds ratio; CI = confidence interval

CMH chisq = Cohran-Mantel-Haenzel chi-squared

Table 3 shows the univariate analyses of categorized insolation testing for dose-response relationships with cognitive function. Two week insolation categorized by 5,000 KJ/m^2^/day showed does-response effects (p = 0.0075). Participants in the lowest category of insolation compared to those in the highest insolation category had 1.36 times (95% CI = 1.08–1.70) the odds of cognitive impairment. When this measure of insolation was modeled as an ordinal variable, each successively lower insolation level compared to the adjacent higher insolation level had 1.06 (95% CI 1.02–1.11) fold odds of cognitive impairment. This study also confirmed prior study findings that there is a dose-response relationship between cognitive impairment and age, income, and education status (Table 3). Depressed participants showed an increased odds of cognitive impairment (OR = 1.90; 95% CI 1.65–2.20). In addition, univariate analyses showed that the seasons of winter (OR = 1.64; 95% CI 1.42–1.90) and spring (OR = 1.46; 95% CI 1.26–1.69) compared to fall gave increased odds of cognitive impairment.

**Table 3:** Crude logistic univariate relationships of cognitive impairment with predictors and final covariates

Characteristics	OR (95% CI)
**Primary Variable of Interest**	

Same day solar radiation (by 5,000 KJ/m^2^/day)	

<10,000 J/m^2^	1.15 (0.96–1.38)

10,000–15,000 J/m^2^	1.02 (0.86–1.21)

15,000–20,000 J/m^2^	0.96 (0.82–1.13)

20,000–25,000 J/m^2^	1.03 (0.88–1.21)

>25,000 J/m^2^	1.00 (Referent)

	CMH chisq p = 0.4346

	

2 week solar radiation (by 5,000 KJ/m^2^/day)	

<10,000 J/m^2^	**1.36 (1.08–1.70)**

10,000–15,000 J/m^2^	1.13 (0.95–1.33)

15,000–20,000 J/m^2^	1.09 (0.92–1.30)

20,000–25,000 J/m^2^	1.01 (0.86–1.19)

>25,000 J/m^2^	1.00 (Referent)

	CMH chisq p = 0.0075

	

**Covariates**	

	

Male	**1.29 (1.15–1.44)**

	

Education	

Less than High School	**4.56 (3.57–5.83)**

High School	**3.26 (2.55–4.17)**

Some College	**1.91 (1.49–2.46)**

College Diploma	1.00 (Referent)

	CMH chisq p < .0001

	

Income	

Less than $20,000 per year	**4.47 (3.46–5.77)**

$20,000 to $35,000 per year	**3.21 (2.49–4.15)**

$35,000 to $75,000 per year	**1.88 (1.45–2.45)**

$75,0000 or more per year	1.00 (Referent)

	CMH chisq p < .0001

	

Age	

Less than 55 years	1.00 (Referent)

55 to 59 years	1.15 (0.89–1.50)

60 to 64 years	**1.30 (1.01–1.68)**

65 to 69 years	**1.80 (1.41–2.29)**

70 to 74 years	**2.36 (1.85–3.02)**

75 to 79 years	**3.11 (2.43–3.99)**

80 or more years	**4.25 (3.30–5.46)**

	CMH chisq p < .0001

	

Region	

Non Belt/Buckle	1.00 (Referent)

Stroke Belt	1.07 (0.95–1.21)

Stoke Buckle	0.91 (0.77–1.06)

	

Season	

Summer	1.00 (Referent)

Fall	0.98 (0.83–1.16)

Winter	**1.64 (1.42–1.90)**

Spring	**1.46 (1.26–1.69)**

	

Depressed	**1.90 (1.65–2.20)**

	

PCS-12 (by 10 unit increase)	**0.82 (0.78–0.86)**

	

Never Used Alcohol	**1.44 (1.28–1.61)**

OR = odds ratio; CI = confidence interval; PCS = Physical Components Summary

CMH chisq = Cohran-Mantel-Haenzel chi-squared

ORs in **bold **have CI's which do not overlap a null value

**Figure 1:**
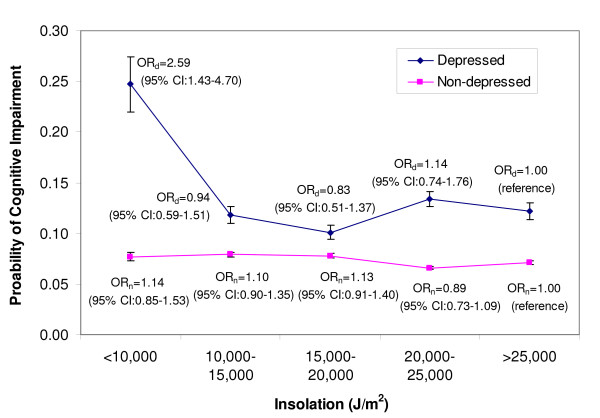
**Predicted Probabilities and Odds Ratios of Cognitive Impairment by Depression Status**.

Due to non-significant (p > 0.10) relationships of high cholesterol, BMI, and smoking with cognitive impairment in crude analyses, these variables were not considered for model-building. Further, population density, diabetes, hypertension, and weekly exercise were dropped from the model for non-significance (p > 0.10) during backwards selection. The final multivariable model included sex, race, education, income, age, region, depression, PCS-12 and alcohol as covariates. Because depression had a significant interaction with sunlight exposure in this model (p = 0.008), the final model included this interaction term and predicted probabilities of cognitive impairment were calculated according to sunlight exposure category and depression status. Figure 1 shows that the predicted probabilities of cognitive impairment for depressed participants are consistently higher than the predicted probabilities of impairment for non-depressed participants. Figure 1 also shows that depressed participants receiving less than 10,000 KJ/m^2^/day of sunlight exposure compared to depressed participants in other solar exposure categories had a significantly higher predicted probability of cognitive impairment. When season was added to the multivariable model, it was significantly related to cognitive function (p < 0.01). Both spring (OR = 1.20; 95% CI 1.01–1.42) and winter (OR = 1.33; 95% CI 1.07–1.67) seasons compared to summer season showed increased odds of cognitive impairment. The relationships between sunlight exposure, depression, and cognitive impairment were unchanged when season was added to the model, giving identical predicted probabilities as in Figure 1.

Relationships between temporal orientation and short-term recall components of the six-item screener did not reveal any significant univariate or multivariable relationships with insolation, indicating that no single component was likely responsible for the relationship (data not shown).

## Conclusion

We found that among participants with depression, low exposure to sunlight was associated with a significantly higher predicted probability of cognitive impairment. This relationship remained significant after adjustment for season. Among participants without depression, insolation did not have a significant effect on cognitive function.

This study adds to the body of literature that shows that environment and lifestyle profoundly affect those who are prone to Seasonal Affective Disorder (SAD) and other types of depression. Studies based on violent homicides, suicides, and aggressive behaviors have repeatedly demonstrated seasonal characteristics, typically with peaks in the spring. These peaks have been associated with sunlight and other climatic variables [18]. Those with SAD have mental states that vary with season, with regular depressions occurring in the winter and remissions in the spring or summer. It is established that these SAD episodes are associated with the shorter daylight hours occurring in winter [19].

The fact that sunlight exposure was associated with cognition in depressed participants supports our hypothesis that the physiological mechanisms which give rise to seasonal depression may also be involved with sunlight's effect on cognitive function. Leonard and Myint, 2006 laid out a paradigm showing how lack of environmental illumination and other stresses might lead to altered serotonin levels, neurodegeneration, depression, cognitive deficits, and ultimately dementia [20]. Both seasonal and non-seasonal depression have been shown to have relationships with environmental illumination [19,21,22]. Theories regarding the body's seasonal cycles, which affect depression and may also affect cognition, are mostly based on the regulation of the body's circadian rhythms by the hypothalamic suprachiasmatic nuclei (SCN) [6,23]. The SCN are modulated by various factors such as body temperature and physical activity, but are in particular modulated by light received by retinal sensors at optimal wavelengths close to sunlight's dominant wavelength of 477 nanometers [23]. The SCN regulate the body's sleep cycle, body temperature, blood pressure, digestion, immune system, and various hormonal systems. Dysfunctional circadian rhythms and sleep disorders, which can occur from inadequate environmental light, have been associated with cognitive deficits [24]. One of the SCN's regulatory functions are their inhibition of the pineal gland from turning serotonin into melatonin during the presence of daytime light [19]. Abnormalities and regulation of both the melatonin and serotonin systems have been found to vary according to sunlight and light therapy in SAD [25,26], bipolar [5] and schizophrenic [27] patients, and even among those without psychiatric diagnoses [28]. Serotonin and melatonin have also been implicated in many mental and cognitive disorders, such as Alzheimer's disease, Parkinson's disease, and sleep disorders [25,29].

Light has been shown to also affect brain blood flow. Cerebral blood flow has specifically been found to improve after phototherapy in pre-term infants [30] and SAD patients [31], and has repeatedly been found to be associated with cognitive functions, such as memory. Inadequate cerebral blood flow has been found to be a likely cause or result of decreasing cognitive functions among those with cardiovascular diseases [32-34], as well as correlated with age-related diseases such as Alzheimer's [35] and non-age related diseases such as Lyme disease [36]. The relationships that serotonin, melatonin, and cerebral hemodynamics have with sunlight, depression, and cognitive function suggest that persons prone to sunlight-related mood disturbances may also be prone to sunlight-related cognitive difficulties.

This study adds to the limited base of knowledge regarding the relationship of weather variables with cognitive function. Studies that have tested the effects of artificial light on cognitive abilities have found that increased light exposure leads to increased alertness and a variety of changes in regional brain activity [37]. In addition, different spectral wavelengths have been found to have differing effects on memory and other cognitive abilities [38]. However, unlike our study, these studies only dealt with immediately acute effects and did not directly examine the effects of natural sunlight. They also have poor generalizability due to using animals or small numbers of human subjects from populations with particular occupations, socio-economic statuses, or ethnicities. We found only two studies that examined the relationship between cognition and sunlight, both of which only dealt with the effects of immediate, short-term exposure. Sinclair et al. (1994) found that increased sunlight exposure was associated with an increase in heuristic processing, which requires memory storage and relevant memory retrieval, but a decrease in systematic processing, a more complicated process requiring analysis and judgment [39]. Keller et al. (2005) found weak positive correlations between sunny days and performance on two measures of cognition, digit span and openness to new information [1]. A major difference between our study and the previous studies is our method of obtaining the participant's exposure to sunlight. The NASA satellite used to obtain the insolation data in this study is able to record data eight times a day as well as provide an accurate characterization of insolation matched to each participant's geocoded home address. This gives superior space and time precision compared to ground sensors used by previous studies. Keller et al. (2005) used barometric pressure as a surrogate for measuring sunny, clear days. Other studies that have not found significant associations between mood or cognition and sunlight in the general population [40] have directly measured insolation using the nearest available ground sensors, which are centered on metropolitan areas. Satellite data allowed us to obtain multiple daily measurements across urban, suburban, and rural areas.

Exposure misclassification exists as a possible limitation of the study. Exposure misclassifications may have taken place if during the two week exposure measurements participants spent a large amount of time in a climate different than the climate recorded by the satellite. This could happen if participants spent large amounts of time indoors or away from their reported home addresses. Also, the daily insolation values were taken by the satellite sensors recorded simultaneously throughout the four different time zones in the U.S. Thus, this point represented different times in the day for different regions of the country. For example, the insolation values used to calculate insolation for participants in the Eastern time zone correspond to 3-hour sampling periods of 1:00, 4:00, 7:00 and 10:00 AM/PM standard time, while for the Mountain time zone the sampling times are 2:00, 5:00, 8:00 and 11:00 AM/PM standard time. However, the relatively short three-hour intervals at which the measurements were taken captures the diurnal cycle well, and the misclassification due to this issue is quite small. It should also be noted that while the relationships found in this study may not apply in younger people (as our study was restricted to those 45 years or older), the participants of the study were recruited from all over the country, with differing demographics, medical factors, and lifestyle factors.

Due to the exclusion of a considerable proportion (27%) of 19,853 enrolled REGARDS participants from the final model as a result of missing values and poor geocoding, we investigated if the excluded participants differed from those with complete information. While sex, education, region, alcohol, age, and depression status of the excluded subjects were statistically different, the proportions of these variables all differed by eight percentage points or less (Table 4). Covariates with larger differences (over 2%) show a disproportionate inclusion into the model of males, those with college diplomas, blacks, non-belt residents, and those that have ever used alcohol. These variables all have known relationships with cognitive impairment and would be the most likely causes of any bias, which might have resulted in underestimating or overestimating the effect of insolation on cognition.

**Table 4:** Final covariates of excluded and modeled participants

Characteristics	Participants in the Final Model	Excluded Participants	p-value
	N (%)	N (%)	

Total	14,474 (73)	5378 (27)	

			

Male	5944 (41)	1887 (35)	<.0001

			

Education			

Less than High School	1670 (12)	798 (15)	

High School	3751 (26)	1525 (28)	<.0001

Some College	3963 (27)	1407 (26)	

College Diploma	5090 (35)	1628 (30)	

			

Income			

Less than $20,000 per year	3071 (21)	623 (22)	

$20,000 to $35,000 per year	3991 (28)	797 (28)	0.42

$35,000 to $75,000 per year	4904 (34)	914 (32)	

$75,0000 or more per year	2508 (17)	484 (17)	

			

Age			

Less than 55 years	1885 (13)	585 (11)	

55 to 59 years	2583 (18)	848 (16)	

60 to 64 years	2759 (19)	1052 (20)	<.0001

65 to 69 years	2654 (18)	1038 (19)	

70 to 74 years	2063 (14)	758 (14)	

75 to 79 years	1443 (10)	653 (12)	

80 or more years	1087 (8)	436 (8)	

			

Black	6291 (44)	2046 (38)	<.0001

			

Region			

Non Belt/Buckle	6802 (47)	2158 (40)	

Stroke Belt	5228 (36)	2074 (39)	<.0001

Stoke Buckle	2444 (17)	1145 (21)	

			

Season			

Summer	5542 (38)	2080 (39)	

Fall	2957 (20)	1116 (21)	0.76

Winter	2891 (20)	1040 (19)	

Spring	3084 (21)	1142 (21)	

			

Never Used Alcohol	4291 (30)	1842 (34)	<.0001

			

Depressed	1605 (11)	653 (13)	0.0047

			

PCS-12 (mean, stddev)	46.2 (10.6)	45.9 (10.5)	0.0449

stddev = standard deviation; PCS = Physical Components Summary

P-values for categorical variables provided from a chi squared test statistic and for continuous variables provided from a pooled t-test statistic calculated for each variable by inlcusion status.

There always remains the possibility of residual confounding. In addition to the imprecision or bias that may be present in any measurement, we could not account for specific psychiatric diagnoses or medicine consumption. Also, environmental temperature may be related to cognitive function, although temperature fluctuations are partially controlled for by season, exercise, cardiovascular factors, and other possible correlates of temperature [41-44]. Eye function is another possible confounder. Specifically crystalline lens transmittance and papillary area have been found affect circadian photoreception, although controlling for age may reduce confounding from these factors. [23]. The interview's time of the day may also have an effect on cognition; however, the sampling method of REGARDS should result in all participants having an equal chance of being interviewed during a given time resulting in similar time distributions at any given variable level [6].

This new finding that weather may not only affect mood, but also cognition, has significant implications and needs to be further elucidated in future studies. That insolation had a relationship with cognitive function but not depression, and that the effect of insolation on cognition is shown among depressed, but not non-depressed participants indicates that insolation may have a relationship with cognition that is independent of, but modified by, depression. It also suggests the possibility that light therapy that is prescribed for SAD may also improve cognitive function. Future studies involving light and other therapies for SAD should include cognitive function as a variable in order to determine relationships with insolation, mood, and cognitive function. Future studies are also needed to demonstrate particular cognitive deficits. The six-item screener was designed to test global cognitive status for large numbers of participants in an easy and efficient manner. While it has adequate sensitivity and specificity as a screening procedure to identify those most likely to have cognitive deficits, it cannot be used to make any particular diagnosis and is limited in its sensitivity to cognitive deficits of small magnitude. In the future, more specific exams and diagnoses can be used to find the specific effects of sunlight on cognitive processes and diseases. We also show that future research regarding treatment and lifestyle should in particular focus on elderly people, since the older a participant is, the more likely the participant is to be cognitively impaired. In addition, research and possibly programs regarding outreach and health education might be targeted to depressives in lower education groups, not only because they are known to have lower access to healthcare in general, but also because they are at a particularly high risk of cognitive impairment. Many of the prior studies have looked at the effects of weather on mood and cognition as seasonal, but the results of this study demonstrate that the effect of season on cognition can be explained by sunlight and other variables. This study also has an interesting finding regarding those without an elevated level of depressive symptoms. We did not find that sunlight meaningfully affected the cognitive abilities of these individuals. However, this lack of a significant finding may be found due to a number of inadequately controlled for indirect behaviors acting as confounders, since there is previous environmental evidence for both season's effects on cognition and environmental illumination's effects on mood and cognition in general populations. Of particular importance, it may be true that those who are non-depressed may spend more time outside, thus receiving a more adequate supply of environmental illumination [17,19,21,22,45-47].

Because cognitive impairment is also associated with other psychological and neurological disorders, discovering the environment's impact on cognitive functioning within the context of these disorders may lead not only to better understanding of the disorders, but also to the development of targeted interventions to enhance everyday functioning and quality of life.

## Abbreviations

BMI: Body Mass Index; CESD: Center for Epidemiologic Studies Depression Scale; CMH: Cochran-Mantel-Haenzel; KJ/m^2^/day: kilojoules per meters^2 ^per day; NASA: National Aeronautics and Space Administration; NCAP: National Center for Environmental Prediction (NCEP); NARR: North American Regional Reanalysis; REGARDS: REasons for Geographic And Racial Differences in Stroke; OR: odds ratio; PST: Pacific Standard Time; PCS: Physical Components Summary; SAD: Seasonal Affective Disorder; SF-12: 12-item Short Form; SCN: the suprachiasmatic nuclei; W/m^2^: Watts/meters^2^.

## Competing interests

The authors declare that they have no competing interests.

## Authors' contributions

SK performed the analysis and drafted the manuscript. LM was a mentor for the methods, statistical analyses, manuscript editing, and data procurement. WC was a consultant for environmental science, manuscript editing, and procured and managed data. DA was a mentor for methods and manuscript editing. VW was a consultant for cognitive function in the REGARDS dataset and manuscript editing. NS was a mentor for the methods, statistical analyses, and manuscript editing.

## Authors' informations

SK is a PhD student in the Department of Epidemiology at the University of Alabama at Birmingham (UAB) and has done work with Marshall Space Flight Center in Huntsville. LM is an Assistant Professor in the Department of Biostatistics at UAB and is also currently working with Marshall Space Flight Center. BC is a scientist working for the Universities Space Research Association and the Marshall Space Flight Center and has extensive experience using satellite data to characterize earth environment variables. VW is an Assistant Professor, works in the Department of Psychology in the School of Medicine at UAB, is the Director of the Dementia Care Research Program, Assistant Director for Translational Research on Aging and Mobility, and has previously used the cohort used in this study for cognitive research. DA is a Professor in and the chair of the Department of Epidemiology at UAB and has extensive experience in cardiovascular and genetic research, an example being the PI of the Genetics of Left Ventricular Hypertrophy: HyperGEN study. NS is an Associate Professor and an environmental and occupational epidemiologist and pediatrician whose research interests include cancer and infectious diseases epidemiology. Her current research activities include epidemiologic studies relating pesticide exposure and suicide, and of workers in the rubber industry, plastics industry, and chemical manufacturing facilities.
